# Nonreferral of Possible Soft Tissue Sarcomas in Adults: A Dangerous Omission in Policy

**DOI:** 10.1155/2009/827912

**Published:** 2009-12-31

**Authors:** Juan F. Abellan, José M. Lamo de Espinosa, Julio Duart, Ana Patiño-García, Salvador Martin-Algarra, Rafael Martínez-Monge, Mikel San-Julian

**Affiliations:** ^1^Department of Orthopedic Surgery, University of Navarra, 31080 Pamplona, Spain; ^2^Laboratory of Pediatrics, University of Navarra, 31080 Pamplona, Spain; ^3^Oncology Department, University of Navarra, 31080 Pamplona, Spain; ^4^Radiation Oncology Division, Department of Oncology, University of Navarra, 31080 Pamplona, Spain

## Abstract

*Introduction*. The aim of this study is to compare outcomes in three groups of STS patients treated in our specialist centre: patients referred immediately after an inadequate initial treatment, patients referred after a local recurrence, and patients referred directly, prior to any treatment. *Patients and methods*. We reviewed all our nonmetastatic extremity-STS patients with a minimum follow-up of 2 years. We compared three patient groups: those referred directly to our centre (group A), those referred after an inadequate initial excision (group B), and patients with local recurrence (group C). *Results*. The study included 174 patients. Disease-free survival was 73%, 76%, and 28% in groups A, B, and C, respectively (*P* < .001). Depth, size, and histologic grade influenced the outcome in groups A and B, but not in C. *Conclusion*. Initial wide surgical treatment is the main factor that determines local control, being even more important than the known intrinsic prognostic factors of tumour size, depth, and histologic grade. The influence on outcome of initial wide local excision (WLE), which is made possible by referral to a specialist centre, is paramount.

## 1. Introduction

Soft tissue sarcomas (STSs) are a heterogeneous group of tumours that derive from mesodermal tissue and can occur anywhere in the body, although most are in the extremities (59%), trunk (19%), retroperitoneum (15%), or head and neck (9%) [1]. STSs are rare, representing only 1% of malignant tumours in adults [2], but are often aggressive neoplasms with the potential to recur after resection and to spread systemically.

The main prognostic factors are intrinsic to the tumour: histologic grade, depth, and tumour size; however, advanced patient age and presence of metastases at diagnosis also affect the outcome [3]. Surgery in the form of wide local excision (WLE) is the primary treatment strategy, the goal being to completely resect the tumour [4]. Local control of STS is largely dependent on such surgery, and so any patient with a lump that might possibly be a sarcoma—a lump bigger than 5 cm, extending down to the fascia, growing in size, or causing pain—should be referred to an appropriate specialist centre, where diagnosis and treatment will be performed in accordance with oncological criteria [5]. In most developed countries, routine referral of patients with such lumps is prescribed by well-defined guidelines. However, in our country, there is no specific protocol in this respect, and consequently, many patients are attended to in nonspecialist centres. The result is that these patients are referred for definitive treatment only after an initial inadequate excision (IIE) has been performed or after subsequent diagnosis of local recurrence. An IIE is also referred to as a *whoops procedure*: the surgeon does not realise that the lump is a sarcoma until receiving the pathologist's report on the material that has already been excised [5].

Patients subjected to an IIE usually need further surgery, which increases the morbidity of the treatment. In addition, there is evidence that insufficient resection increases the risk of local recurrence [6], and it is well established that local recurrence increases the risk of further local events. There is, however, little published information about the local recurrence rate in *whoops* cases and even less on survival rates. The aim of this study is to compare outcomes in three groups of STS patients treated in our specialist centre, patients referred after a *whoops* procedure, patients referred after a local recurrence; and patients referred directly to our centre for a definitive diagnosis.

## 2. Patients and Methods

We reviewed all patients with a minimum follow-up of two years who had been treated for STS in our hospital between 1983 and 2006. We excluded cases of extraskeletal Ewing's Sarcomas, of STS when located anywhere other than in the extremities, and of patients with metastases at the time of diagnosis.

Patients were assigned to one of the following three groups: group A, the virgin STS group, comprised those who had been referred directly to and first diagnosed at our hospital; group B, *whoops* cases, included patients who had been referred to us immediately after an IIE; and group C, the local-recurrence group, included patients referred after having one or more local recurrences.

In all cases, all resected specimens had been examined histologically and the tumour had been evaluated as high or low grade. For group B patients, findings of residual tumour had been noted. From our records of each patient, the following data at the time of diagnosis were collected: gender, age, and tumour characteristics (size, depth, and histologic grade). For group C patients, we also assessed depth and histologic grade of the primary tumour; in most cases of group C, it was not possible to determine the primary tumour size. In accordance with the criteria of the American Joint Commission on Cancer Staging of STS [7], we regarded tumours of 5 cm or less as small and those of over 5 cm as big; tumours above the fascia were regarded as superficial and those invading or beneath the fascia were regarded as deep; neoplasms that were well- or moderately well-differentiated as low grade and those that were poorly- or un-differentiated as high grade. For patients who had been previously operated on at another hospital (groups B and C patients), we noted any evidence that indicated disregard of basic oncologic surgery rules on the part of the surgeon, such as scar orientation.

Outcomes were analysed in terms of disease-free survival (DFS) and overall survival. Other outcome events, such as local recurrence and distant metastases, were not assessed. In this context of outcome events, for group C patients, we use the term *local recurrence* to refer to any recurrence after the surgical treatment at our institution (as opposed to the recurrence that led to referral).

### 2.1. Statistical Methods

The Chi-square test was used to compare phenotype frequencies between groups (diagnostic categories; tumour grade, depth, and size; and metastasis/relapse status). A multivariate survival analysis (stepwise Cox regression) including prognostic factors (depth, grade, and size) between groups A and B was performed.

Overall survival and disease-free survival data were expressed as Kaplan-Meier curves, and groups were compared by means of the logrank test (Mantel-Cox test). The statistical analysis was performed with the SPSS software v15.0 (SPSS, Statistical Package for the Social Sciences, Chicago, IL; USA). In all cases statistical significance was defined as *P* < .05.

## 3. Results

### 3.1. Patients

We treated a total of 236 STS patients between 1983 and 2006. Of them, 209 involved tumours in the extremities. We excluded 25 patients with metastases at diagnosis and 10 cases of extraskeletal Ewing's Sarcoma. The cases analysed then numbered were 174.

The mean age of these patients was 43.74 years, with a standard deviation (SD) of 18.8 years. Mean follow-up was 91.95 months (SD, 80.16 months), with no differences between the three groups and a minimum follow-up of two years. 

**Figure 1:**
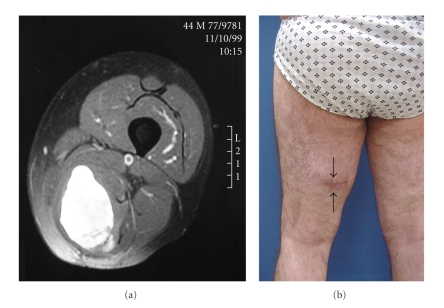
(a) Axial thigh T2-MR showing a hyperintense mass in the posterior aspect of the thigh. (b) Clinical image showing the wrong incision (black arrows) performed to excise the lump with the preoperative diagnosis of a benign lump. Histologic analysis established that the mass was a sarcoma; the excision had thus been a *whoops* procedure.

**Figure 2:**
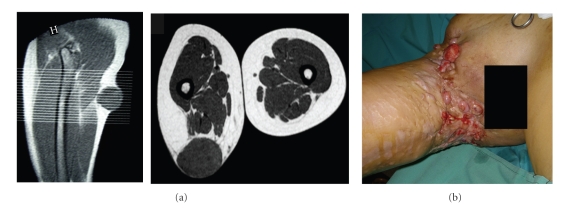
(a) An example of the few patients in group C for whom we have data about the primary lump. Thigh T1-MR, showing a subcutaneous lump (Malignant Fibrous Histiocytoma) in the groin. The mass was excised, in another hospital, without regard for basic oncologic rules. (b) Clinical image showing how we first encountered the patient, who had suffered multiple local recurrences.

Of the 174 cases, 57% were in group A, 22% were in group B (Figure 1), and 21% were in group C (Figure 2). Basic group characteristics are shown in Table 1.

### 3.2. Treatment

Patients were treated by surgery: radical excision with curative intention. Amputation as a primary therapeutical procedure was not performed. Wide local excision was complemented by radiation therapy and chemotherapy as standard treatment of deep, big, and/or high-grade STS. Of such patients, only 30% did not receive chemotherapy, because of a contraindication to the procedure (such as cardiologic disease). We now complement external radiotherapy with brachytherapy, but, in the patients treated between 1983 and 2001, we used an intraoperative boost dose of radiotherapy to the same end. 

Histologic analysis detected malignant cells in all the specimens from patients in groups A and C and residual tumour cells in 40% of specimens from group B cases.

Analysis of previous operations, biopsies, or excisions suggested that oncologic surgery rules were frequently disregarded: in 39% of group B patients and 30% of group C patients. The most frequent indicator of disregard was an inappropriate transversal approach incision (Figure 1(b)).

### 3.3. Outcome

**Local Recurrence**

Overall, local recurrence occurred in 21% of patients. The rates in groups A and B were 10% and 13%, respectively (*P* = .608); in group C the rate was 59%. This difference, A + B versus C, was statistically significant and was not related with depth and histologic grade (Table 2).

**Metastatic Disease**

Overall, 27% of the patients suffered metastatic disease: 22% in group A, 16% in group B, and 51% in group C (Table 3). The higher rate for group C patients was statistically significant and was independent of both depth and grade of the tumour.

**Survival**

**Figure 3:**
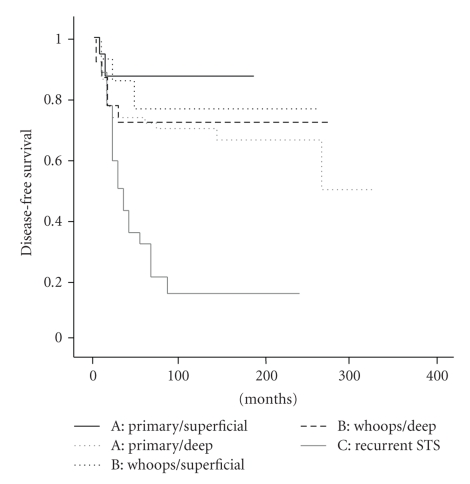
Kaplan-Meier curve of disease-free survival rates. Groups have been stratified according to tumour depth.

The rate of DFS was significantly lower in group C patients, 28%, than those in groups A and B patients, 73% and 76%, respectively (*P* < .0001). As shown in the Kaplan-Meier curves of Figure 3, when considering only cases with superficial STS, the highest disease-free survival rates were for patients in group A. Multivariate analysis between groups A and B showed that only tumour size statistically influenced both overall and disease-free survival (*P* = .024).

**Figure 4:**
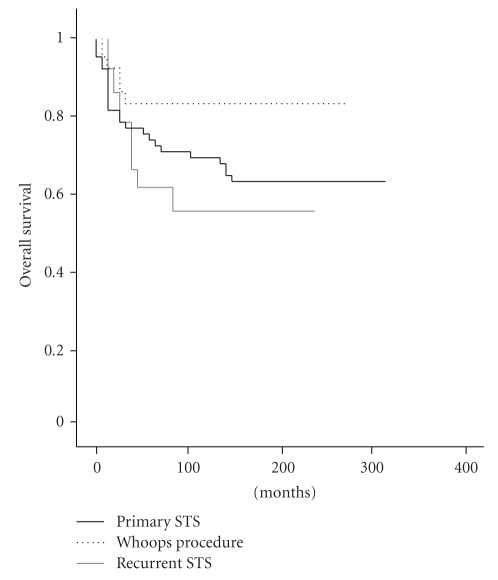
Kaplan-Meier graph showing overall survival comparing the three groups.

With regard to overall survival, the mean for the three groups was 69.9%, with no significant differences between the groups (*P* > .05) (Figure 4). The overall survival of group B patients is apparently, but not statistically, higher. As with DFS, this is because the *whoops* STS were mostly subcutaneous (Table 1).

## 4. Discussion

The current view is that STS treatment in a referral centre, where diagnosis and treatment follow oncologic rules, improves results in terms of both quality of life and survival [3]. However, because soft tissue sarcomas (STSs) are rare—on average a family doctor will see just one case for every 24 years of practice [8]—malignancy is not always suspected, with the consequence that referral is delayed, or the patient is treated in a nonspecialist centre.

The retrospective series of patients we present and analyse here does not provide a representative sample of STSs. This is indicated, for example, by the fact that between 1980 and 2006 our centre treated over 1000 bone sarcomas but less than 300 STSs in the extremities.

Without a biopsy, an STS, especially if it is subcutaneous and small, is sometimes misconstrued as a benign mass and surgically excised without the adequate margins for a malignant mass. Histologic study of the resected lump overturns the preoperative diagnosis and establishes the previously unsuspected diagnosis of sarcoma. Such an incident is known as *inadequate initial excision* (IIE) and a *whoops procedure* [5]. In the 1990s, between 19% and 53% of the new patients seen in sarcoma centres were referred after an IIE [9–11].

To ensure early diagnosis and management of all suspected STS cases, developed countries such as the UK drew up referral guidelines [12] according to which any patient with a lump suspected of being an STS or with a recurrence of a previously excised lump must be referred to a specialist centre with an appropriate multidisciplinary approach; a lump should be suspected of being an STS if it is bigger than 5 cm, deeper than the fascia, growing in size, or causing pain.

In our practice, we still see patients referred after *whoops* procedures. These patients are often distressed by the unexpected diagnosis and, like their physicians, concerned about the prognosis and further management of the disease.

Wide local excision (WLE) is the correct primary treatment strategy for extremity STS. The overall approach to treatment should be multidisciplinary: with a specialist sarcoma surgeon, radiologists, pathologists, and clinical oncologists [3]. Recent multimodal strategies combining surgery and radiotherapy, complemented or not with chemotherapy, have enabled WLE to replace amputation as standard treatment without increasing local recurrence rates or decreasing overall survival [13]. In recurrent STS, some authors consider that amputation may decrease the local recurrence rate [14, 15]; benefits in overall survival have not been reported.

For patients with nonmetastatic STS, overall survival is 50%–70% [4, 16]. There are three main prognostic factors: tumour size, histologic grade, and depth. In addition, some authors have recently found advanced patient age to be a prognosis factor [3].

In our study we categorized patients into three groups to determine how an initial inadequate treatment of an STS affected the outcome. We found that patients diagnosed in and treated at our centre (group A) and patients referred immediately after an IIE (group B) presented similar results in terms of local recurrence rate, metastasis rate, disease-free survival, and overall survival (Tables 2 and 3, Figures 3 and 4). Given that some of the patients were initially treated in another centre, data on surgical margins were not always available. This important factor regarding local control has not been therefore considered in the present analysis.

Paradoxically, patients treated after an initial *whoops* event had better outcomes than those who were diagnosed and treated directly in our institution (Figure 3). This finding, also reported by Lewis et al. [9], is explained by the fact that big, deep lumps are usually referred whilst the small, subcutaneous STSs are more likely to be misconstrued as benign and only to be discovered after a *whoops* event. In our series, 83% of virgin cases and 60% of *whoops* cases were deep tumours. When survival is calculated for primary tumours of equal depth, our virgin-STS patients showed better outcomes than the *whoops* patients (Figure 3).

Our third group was for patients with one or more recurrences, with or without a *whoops* event, prior to treatment at our centre. Surgical treatment of these patients was complemented with an established combined treatment including perfusions of isolated limbs with tumour necrosis factor and melphalan [17] or perioperative high-dose-rate brachytherapy [18, 19]. The local recurrence and metastasis rates for group C (after treatment in our centre) were worse than the rates for groups A and B (Figure 3). This was the case despite the proportion of low grade STSs at primary presentation being higher in group C (Table 1). The results of group C are shown without statistical comparison with groups A and B, because they represent a different clinical situation (primary versus relapsed tumours). The poor outcome in these patients could be explained by supposing that the initial STSs were more aggressive, but we believe that the real cause of the low disease-free survival is related to inadequate initial surgical excision. There are two reasons to justify this position. First, one third of the patients in this group presented scars indicative of wrong incisions (Figure 1(b)). The implied disregard of basic oncologic rules suggests that the surgeon was not used to treating malignant lesions, and that it is unlikely that WLE was performed. Second, overall survival in this group is above 55% (Figure 4): such high overall survival is not compatible with a supposition of STS aggressiveness.

The big question when faced with an IIE patient is whether further surgery is necessary. We believe that the answer is yes. Previous reports show that unplanned excisions of STS tend to be incomplete [6, 10, 20–23], and residual tumour cells were commonly found (35%–77% of cases). Our results support these findings: Forty percent of our IIE patients showed evidence of residual disease. Wide excision of residual tumour after an IIE is, therefore, imperative to achieve local control of the disease. In our study, *whoops* patients who we reoperated on with wide excision had similar survival outcomes to patients who were diagnosed and treated directly at our centre. Good results after reexcision of an IIE have been reported for other specialist centres [5, 6, 20, 22]. However, to our knowledge this is the first study to compare a single centre's results with IIE reexcisions and with both direct treatments and treatments of local recurrence secondary to an IIE. Our results indicate that initial WLE is the main factor determining local control; this factor has a huge influence on the outcome, even greater than that of the intrinsic prognostic factors (histologic grade, tumour size, and depth).

Although survival after wide reexcision in a *whoops* patient is similar to that for a direct WLE patient, the morbidity rate is higher for the former because, first, at least one additional operative intervention (the reexcision) is needed; second, a greater volume of tissue is resected; third, a larger field is radiated; and fourth, soft tissue coverage procedures are more frequent [24]. In our study, 95% of reexcised *whoops* patients needed adjuvant treatment. Therefore, to avoid complications, it is important that all patients with suspicious lumps be referred to a specialist centre. The situation with STS is the same as that asserted by Wafa and Grimer with regard to bone sarcoma [25]: there is no role for occasional surgeons; the first surgical excision is critical in the prognosis and outcome for these patients, and thus they must be treated in specialist centres.

## 5. Conclusions

A patient with a lump which in any way raises a suspicion of STS should be referred to a specialist centre, where a multidisciplinary team with good experience of such infrequent lesions can assess the patient and determine the best treatment.

The unplanned excision of a malign mass, the *whoops* procedure, should be immediately followed by wide reexcision, which in our study resulted in survival rates similar to those for patients operated on directly with WLE. Reoperated patients suffered more radiotherapy related complications and a general increase in the morbidity rate. Patients presenting a local recurrence after an IIE had worse outcomes, especially in terms of disease-free survival.

Initial wide surgical treatment is the main factor that determines local control, being even more important than the known intrinsic prognostic factors of tumour size, depth, and histologic grade. The influence of initial WLE on outcome is paramount. However, these conclusions should be interpreted with caution, because the number of patients in each group is relatively small and important differences might be undetected.

## Figures and Tables

**Table 1:** Data on size, depth, and grade in the different STS groups considered.

	N	Age (SD)	Size (%)		Depth (%)		Grade (%)		
	174	43.74 (18.7)	≤5	>5	^1^Sup.	Deep	Low	High	
Group A	99	46.17 (18.4)	14 (14)	85 (86)	17 (17)	82 (83)	34 (34)	65 (66)	
Group B	38	35 (18.2)	16 (42)	22 (58)	15 (39)	23 (61)	12 (32)	26 (68)	
A versus B *P*-value^2^		.002	<.001		.006	.759		
Group C	37	46.22 (17.9)	^3^NA	NA	2 (5)	35 (95)	18 (49)	19 (51)	
A + B versus C *P*-value		.005	—		.001		.230		

^1^Sup.: superficial; ^2^the statistical analysis does not include group C; ^ 3^NA: not available.

**Table 2:** Characteristics of the STS in the different groups according to recurrence.

	Recurrence (%)	Depth (%)		Grade (%)	
		Superficial	Deep	Low	High
Group A	10 (10)	0 (0)	10 (100)	3 (30)	7 (70)
Group B	5 (13)	2 (40)	3 (60)	2 (40)	3 (60)
Group C	^1^22 (59)	10 (46)	22 (54)	11 (50)	11 (50)
A versus B *P*-value^2^	.608				
A + B versus C *P*-value	<.0001				

^1^This value refers to the rate of local recurrence after treatment in our institution.

^2^The statistical test does not include group C.

**Table 3:** Characteristics of the STS in the different groups according to metastasis.

	Metastasis (%)	Depth (%)		Grade (%)	
		Superficial	Deep	Low	High
Group A	22 (22)	6 (27)	16 (73)	5 (23)	17 (77)
Group B	6 (16)	2 (33)	4 (67)	0 (0)	6 (100)
Group C	19 (51)	10 (50.0)	18 (51.4)	8 (42)	11 (58)
A versus B *P*-value^1^	.403				
A + B versus C *P*-value	<.001				

^1^The statistical test does not include group C.
